# Global trauma: the great divide

**DOI:** 10.1051/sicotj/2015019

**Published:** 2015-07-21

**Authors:** Jayanth Paniker, Simon Matthew Graham, James William Harrison

**Affiliations:** 1 Department of Orthopaedics, Countess of Chester Hospital Liverpool Road Chester CH2 1UL UK

**Keywords:** Accidents, Traffic, Health resources

## Abstract

Road trauma is an emergent global issue. There is huge disparity between the population affected by road trauma and the resource allocation. If the current trend continues, a predicted extra 5 million lives will be lost in this decade. This article aims to create an awareness of the scale of the problem of road trauma and the inequality in the resources available to address this problem. It also describes the responses from the international organisations and the orthopaedic community in dealing with this issue. The International Orthopaedic community has a unique opportunity and moral obligation to play a part in changing this trend of global trauma.

## Introduction

Road traffic accidents are the single biggest cause of injuries and injury-related mortality, accounting for a quarter of all injury deaths [1]. Worldwide, an estimated 1.3 million people are killed in road traffic crashes each year and as many as 78.2 million are injured [2]. Projections indicate that these figures will increase by about 65% over the next 20 years, unless there is a new commitment to prevention [2]. This effect of projected increases will be greater in low- and middle-income countries, as they currently account for over 90% of all road traffic injury deaths [2].

The problem of road trauma and its growing trend in the low- and middle-income countries is not a new problem and it has previously been described as a neglected global epidemic [3]. The course of global health problems, like HIV and malaria, has been altered with concerted international effort. Progress in addressing road trauma however has been slow and the predicted trend of this epidemic has not changed.

The aim of this article is to create an awareness of the scale of this problem and to emphasise the geographical distribution of road trauma whilst highlighting the disparity in the resources available to address this problem.

## The problem

Road trauma is a major cause of both death and disability worldwide. There is an associated social and economic impact. The resources available to address this problem effectively vary greatly across the different regions of the world.

### Injury

**Figure 1.**
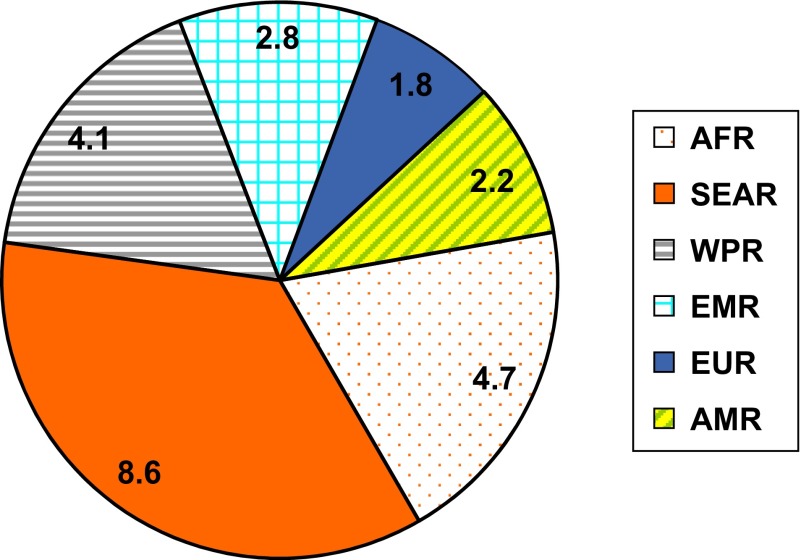
Chart depicting figures for the road traffic injuries (in millions) for the WHO regions (AFR – Africa, SEAR – South East Asia, WPR – Western Pacific, EMR – Eastern Mediterranean, EUR – Europe, AMR – Americas).

Worldwide road traffic accidents account for an estimated 50 million injuries [4]. These are the reported injuries and certain sources estimate the figure to be around 78.2 million [2]. There is huge disparity in the injury figures for the different WHO regions. The South East Asia Region (SEAR) and Africa Region (AFR) which comprise of low- and middle-income countries account for over 50% of the injuries (Figure 1).

Injuries sustained by victims of a road traffic crash vary in type and severity. There are notable differences between road user groups. Pedestrians and two-wheeler users are at greater risk than vehicle occupants and bear the greatest burden of injury. This is especially a problem in low- and middle-income countries due to poor road safety measures and the greater variety and intensity of traffic mix.

Recent, population-based analyses of road traffic injuries in urban Tanzania [5] and urban Ghana [6] have demonstrated that these injuries are a major source of disability in these developing countries. A review of studies in low-income and middle-income countries [7] revealed that road traffic-related injury accounted for between 30% and 86% of trauma admissions in these countries. The review further found the following:

- road traffic injury patients comprised between 13% and 31% of all injury-related attendees in hospitals,
- road traffic injury patients represented 48% of bed occupancy in surgical wards in some countries,
- road traffic injury patients were the most frequent users of operating theatres and intensive care units.

### Deaths

Worldwide, over 1.3 million people die from road traffic accidents [2] every year. This is projected to increase by 66% to 2.34 million by 2020 [8]. These trends are however divergent, with a defined global distribution. In the low- and middle-income countries, there will be a projected increase of over 80% whereas in the high income countries there will be a predicted fall of about 30% [8].

**Figure 2.**
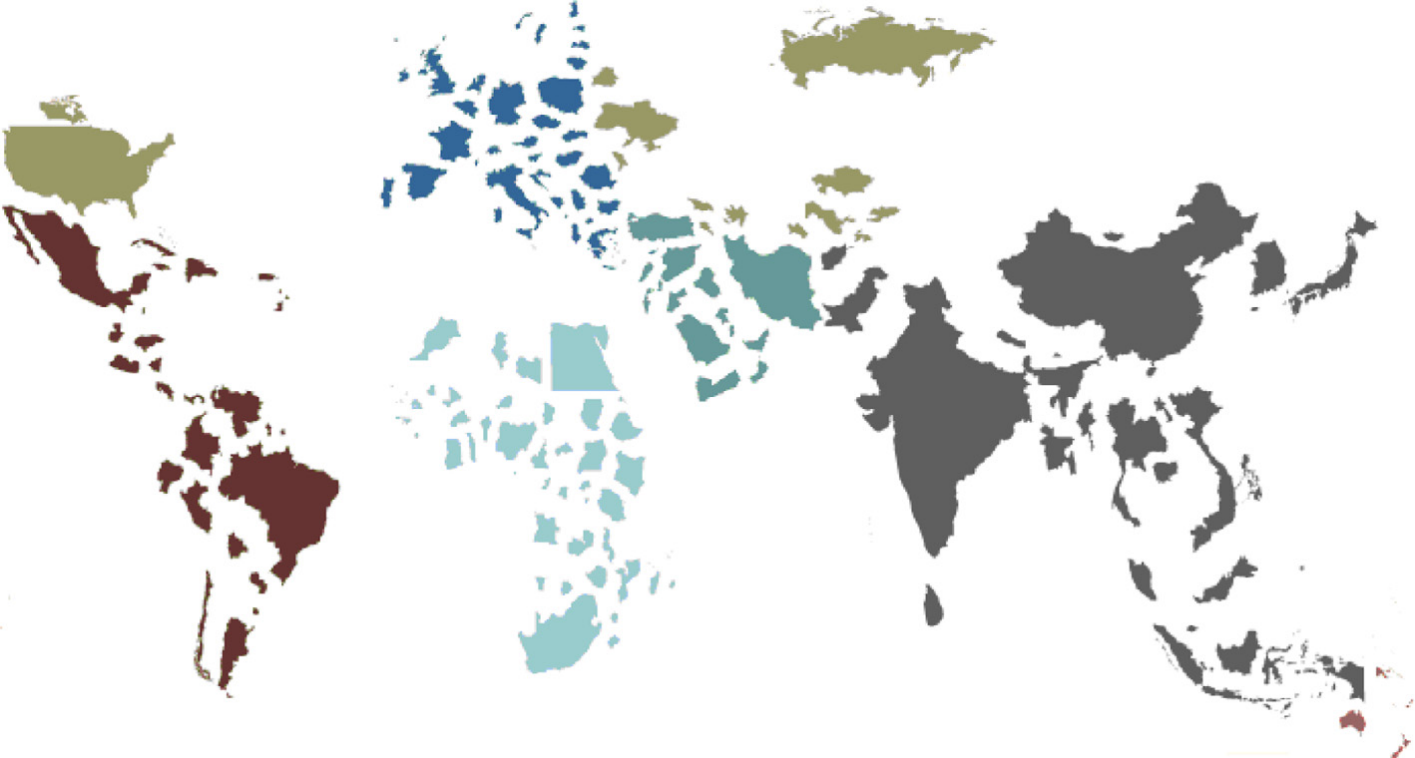
World map depicting countries in a size relative to the number of road traffic deaths (note how small Europe & USA appear).

Currently, low-income and middle-income countries account for about 90% of the deaths from road traffic accidents [2]. There are regional differences in mortality with the WHO SEAR region alone accounting for about 35% of global road traffic mortality (Figure 2). A recent survey of traumatic injuries from Sierra Leone, Africa found that even though traffic injuries accounted for only about 9% of all injuries it was the leading cause of injury-related deaths [10].

Road traffic deaths tend to occur most commonly in younger males. Globally, the road traffic injury mortality rate for males is almost three times higher than that for females. Also over 50% of the global mortality due to road traffic injury occurs among young adults aged between 15 and 44 years [2].

### Social and economic impact

The impact of road traffic injuries on the economy is estimated to be about 1% of Gross Domestic Product (GDP) for low-income countries, 1.5% for middle-income countries and 2% for high-income countries [11]. These figures are paradoxical and indicate the higher resource allocation to injured parties in wealthier countries, where numbers of injured are much fewer. Globally the cost of road trauma is estimated to be approximately US $518 billion per year [11]. This WHO figure is an underestimation, as, in the United States alone the figure totalled more than $400 billion for the combined economic burden of medical treatment and lost productivity [12].

Aside from the heavy burden placed on global and national economies, road traffic accidents have a big impact on the families involved. Around 60% of the total number of disability-adjusted life years (DALYs) lost as a result of road traffic accidents occur among young adults aged between 15 and 44 years [1]. 73% of these were young adult males [1]. These young adults are often the bread-winners and their loss drives many families into poverty.

**Figure 3.**
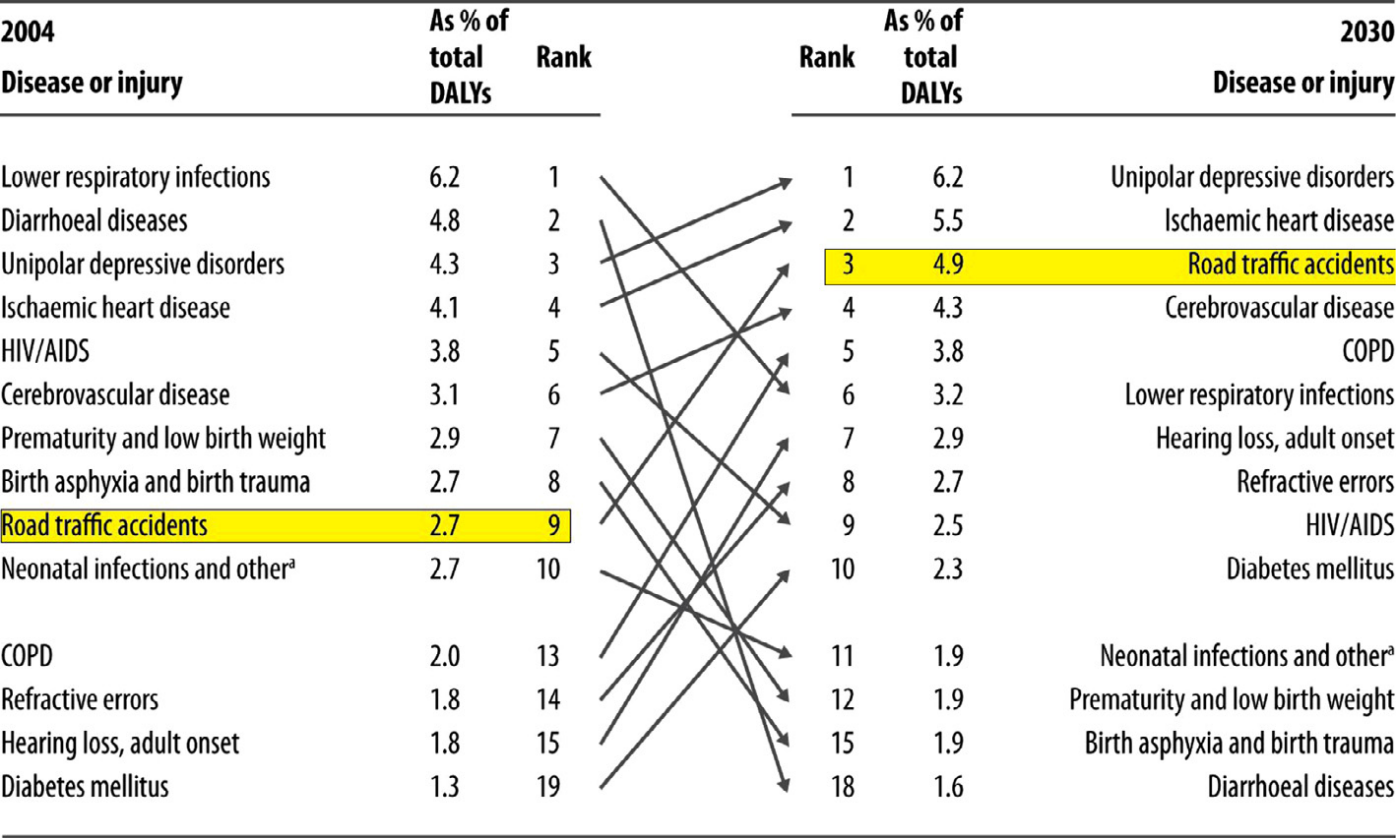
Table comparing the DALYs ranking between 2004 and 2030 [4].

In 2012, road traffic injuries were the eighth leading cause of DALYs lost, up from tenth place in 2000, but by 2030, they are predicted to become the third leading cause of DALYs lost globally (Figure 3) [13]. DALYs lost will increase worldwide from 34.3 million to 71.2 million (representing 5.1% of the global burden of disease) [13]. However, in low- and middle-income countries this is an even bigger problem, as over 90% of the global road traffic-related DALYs lost, occurs in these countries. Furthermore in such countries road trauma is predicted to become the second leading cause of DALYs lost [13].

There appears to be a direct relationship between road traffic deaths and per capita income. The trend is an initial increase in road traffic deaths with per capita income, which reaches a peak, and then declines. The per capita income at which road traffic death peaks is estimated at $8600. Beyond this level of income the rate of road traffic deaths declines [14]. This is relevant, as it is in the middle-income countries that the majority of the world population lives and where there is a greater burden of disease from road traffic injuries and deaths (Figure 2). For example, in 2013, the per capita income of India was $5350 and as shown in Figure 2 it accounts for a large proportion of road traffic deaths [15].

### Resources

In 2011, the world spent a total of US $6.5 trillion on health; however, the geographical distribution of financial resources for health is disproportionate [16]. There is a clear 20/80 divide with 34 Organisation for Economic Co-operation and Development (OECD) countries which make up less than 20% of the world population spending over 80% of the world’s resources on health. This is in contrast to less than 20% of health resources being spent on the remaining 80% of the world population [16].

The disparity is even more in certain regions of the world. For example, the WHO regions, Africa (AFR) and South East Asian Region (SEAR), which account for the largest share of the global burden of road trauma (over 50% of DALYs lost) and 38% of the world’s population, spend only 2.5% of the global health resources [16].

**Figure 4.**
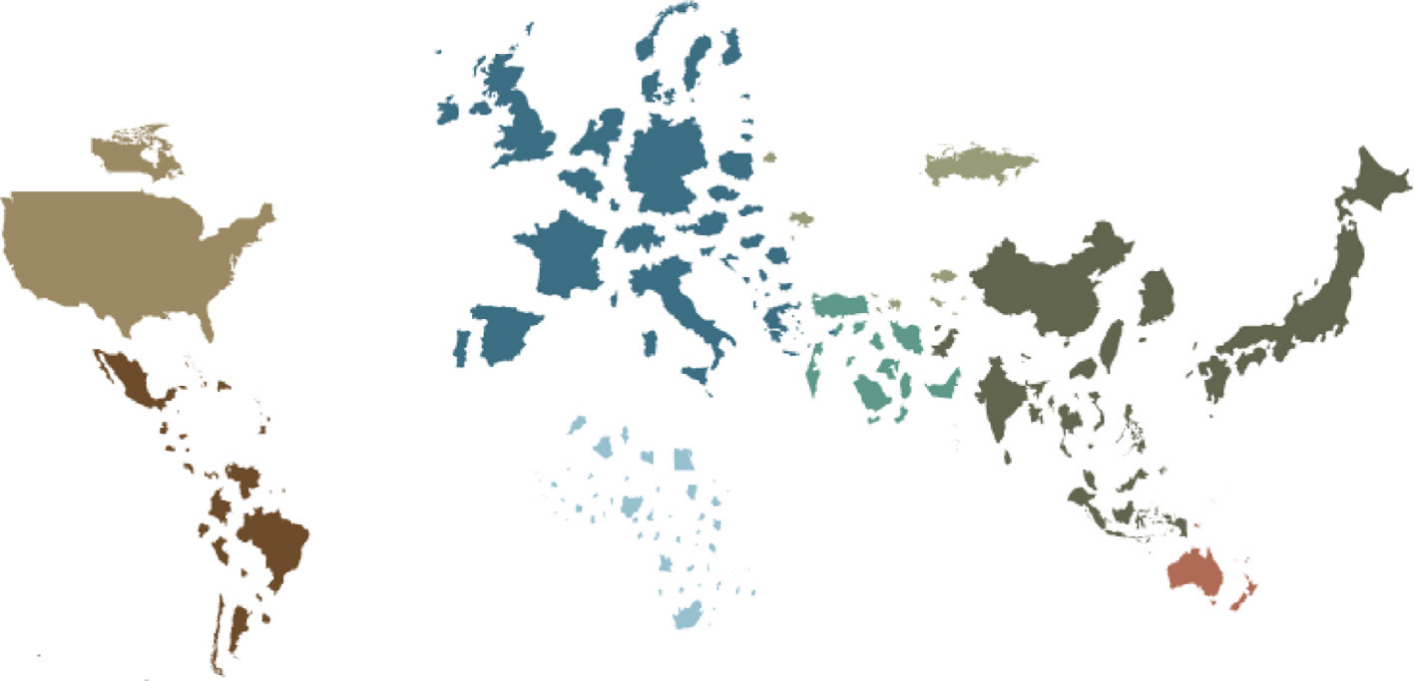
World map depicting countries in a size relative to their GDP (note the reversal of size distributions shown in Figure 2).

Though there is an obvious link between the GDP of a country (Figure 4) and road trauma, it is not the only reason for the variation. The percentage of GDP allocated for health spending in 2009 also varied widely, ranging from 2.1 in Myanmar to 18.9 in the Marshall Islands. There are also large regional differences, with the South East Asian Region spending only 3.8% of its GDP on health compared with the region of the Americas spending 14.4% of their GDP on health [16]. The health expenditure per capita also varies greatly. In 2013, it was $9146 for the United States compared with only $61 for India [15].

In contrast to the economic and social impact caused, little money is invested in preventing road traffic injuries. Other current global health issues like HIV and malaria receive far larger investment in global research and funding. This is having a direct effect in reducing the impact of these diseases. By 2020, road traffic injuries are predicted to be the 3rd leading contributor to the global burden of disease and injury, overtaking health issues which receive much greater investment [17] (Table 1).

**Table 1.** Estimated global research and development funding for selected topics [3].

Disease or injury	US $ millions	1990 DALYs ranking	2020 DALYs ranking
HIV/AIDS	919–985	2	10
Malaria	60	8	24
Diarrhoeal diseases	32	4	9
Road traffic crashes	24–33	9	3

There is evidence that the discrepancy in health resource allocation impacts directly on road trauma mortality figures. For example, one study looked at the mortality rates for all seriously injured adults in countries at different economic levels. The mortality rate rose from 35% in a high-income setting to 55% in a middle-income setting, to 63% in a low-income setting [18]. A similar study looking at the mortality rate of moderately injured patients who reached a hospital showed a mortality rate of 6% in a hospital in a high-income country compared with 36% in a hospital in a low-income country [19].

## The response

Decreasing the global burden of road trauma needs a multifaceted approach. This should include

- increased awareness with improved trauma data collection,
- involvement from international organisations and governments,
- individual and small-scale response.

### Awareness

The WHO has recognised road trauma as a significant global health issue and has declared this decade as the Decade of Action for Road Safety (2011–2020). This is aimed at increasing awareness and raising the profile of the preventability of road traffic accidents. It was launched in more than 100 countries, with one goal: to prevent 5 million road traffic deaths globally by 2020.

HIV, malaria and TB are largely problems of the low- and middle-income countries, yet there is greater awareness of its impact. As a result high-income research and development organisations have applied massive funding. In the case of HIV the perceived impact and fall in global DALY rating is encouraging. Similar investment in addressing road trauma is likely to be equally or more cost effective.

### International involvement

The WHO is working with other organisations to help reduce the global impact of road trauma. Recently they collaborated with the International Association for the Surgery of Trauma and Surgical Intensive Care (IATSIC) to launch the Essential Trauma Care Project. The project seeks to define what essential trauma treatment services should realistically be made available to every injured person worldwide.

WHO projects that have shown promising results include the “Village University” concept, which involves training local communities in pre-hospital life-support methods and delegating life-saving skills to non-doctors. This has resulted in a reduction of trauma mortality from 40% to 8% in northern Iraq and northwest Cambodia [20].

Organisations like the Red Cross, the World Bank and international road safety organisations like the Fédération Internationale de l’Automobile (FiA) foundation are working with local governments to help improve road safety and reduce road traffic deaths and injuries. These measures will include road safety education, enforcement and infrastructure.

The UK Department for International Development (DFID), which manages the UK’s overseas aid programme, has joined the World Bank Global Road Safety Facility which again works towards reducing road deaths.

Several orthopaedic organisations are already working to reduce trauma-related deaths and injury through different approaches. Orthopaedic Overseas and Surgical Implant Generation Network (SIGN) send volunteer orthopaedic surgeons to train and educate local health providers. SIGN also develops and distributes orthopaedic trauma implants suited for areas with limited facilities. Institute for Global Orthopaedics and Traumatology (IGOT), and World Orthopaedic Concern provide support through education, training, research and advocacy. Société Internationale de Chirurgie Orthopédique et de Traumatologie (SICOT) provides access to online training through Webinars and SICOT Global Network of Electronic Learning (SIGNEL) and provides grants for orthopaedic surgeons from developing countries through SICOT Foundation programmes. Though involvement of these organisations is having an effect, the impact is still relatively small.

### Small-scale response

The UK postgraduate specialty training programme currently supports the *Global health partnerships: the UK contribution to health in developing countries* (*2007*) [9]. This is aimed at allowing trainees to take time out of their training to work in developing countries. This experience is invaluable both to the countries where they work but also to the trainee. Trainee involvement in development of trauma services and training should be encouraged.

Many UK hospitals have partnerships with aid agencies or hospitals in low- and middle-income countries. Most of the funding and training provided by these hospitals is focussed on relevant issues like child and maternal health, with promising results. There are only a few partnerships that are focussed on trauma. The COOL (COSECSA [College of Surgeons of East, Central & Southern Africa] Oxford Orthopaedic Link) Project, which has trained 1550 frontline health workers across 10 COSECSA countries in trauma management, is one example. The Paired Institutional Partnerships organised by the Tropical Health and Educational Trust has created numerous partnerships between UK hospitals. The South Devon Healthcare NHS foundation Trust has linked with the Nanyuki Hospital in Kenya and focusses their resources on reducing the mortality and morbidity from traumatic injuries. If more of the resources and training were focussed on road trauma it could result in a significant reduction in the morbidity and mortality in these countries.

**Figure 5.**
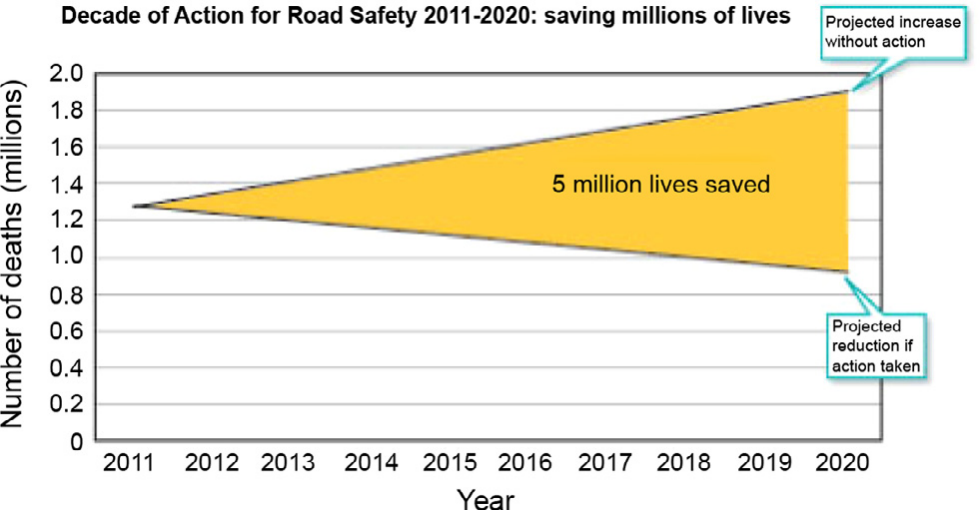
WHO projected trend of road deaths to 2020.

The WHO predicts that if action is taken 5 million lives, 50 million serious injuries and US 5 trillion dollars can be saved in this decade (Figure 5). Only with a concerted multifaceted approach can this global problem be addressed. Orthopaedic surgeons can have a direct impact by volunteering, by organising paired partnerships and by lobbying the multinational orthopaedic companies and organisations to intervene. The International Orthopaedic community has a unique opportunity and moral obligation to play a part in changing the trend of global trauma.

## Conflict of interest

Authors JP, SMG and JWH certify that they have no financial conflict of interest (e.g. consultancies, stock ownership, equity interest, patent/licensing arrangements, etc.) in connection with this article.

None of the authors have any conflict of interest.
